# Investing in Women Trainees: Building a Women in Medicine Group at an Academic Institution

**DOI:** 10.2196/47783

**Published:** 2023-05-31

**Authors:** Lydia Zhong, Koeun Lee, Maria Q Baggstrom, Rakhee K Bhayani

**Affiliations:** 1 Division of General Medicine Washington University School of Medicine St Louis, MO United States; 2 Division of Oncology Washington University School of Medicine St Louis, MO United States

**Keywords:** gender equity, graduate medical education, women in medicine, trainee wellness, women, gender gap, inequity, medical training, medicine

## Abstract

Given the importance of proactively supporting women trainees in medicine to address gender inequities, we draw on the experience of a well-established professional development initiative to provide a framework for other institutions seeking to create similar trainee-focused programs.

## The State of Women in Medicine

The long-standing gender gap in medicine remains an urgent and complex issue. Gender-based inequities can be found at the earliest time points in medical training, such as the residency application and selection process [1-3]. Gender inequities persist throughout the course of an academic medical career; these include longer periods between promotions [4], less department leadership representation [5], lower pay and more unpaid work for equal productivity and practice type [6], and continuing bias and discrimination [7]. Women physicians are also more likely to have caretaking responsibilities in addition to their careers [8].

There have been several advances in academic medicine that address gender inequities, and many more suggestions have been proposed: regulating trainee working hours [8], introducing inclusive and respectful work environments [9], promoting women to positions of leadership [10], and more flexible leave policies [11]. Institutional programs such as the initiation or implementation of women in medicine groups are one of the measures taken to make progress. However, most interventions have been targeted for faculty with few intended to meet the needs unique to residents [12,13]. Here, we describe our experience with designing and sustaining an intervention that addresses the need for resident support as an effective approach that can be adopted by other institutions resolving to advocate for gender equity in medicine.

## A Potential Solution

In 2014, a structural approach to promote women in medicine by supporting trainees was undertaken at the Department of Medicine of Washington University School of Medicine. The Forum for Women in Medicine (FWIM) was created to organize a series of workshops for women trainees to meet their needs. An initial needs assessment evaluated gaps in programming and guided the development of FWIM. Over 50% of those who completed the survey had a significant other who was also a physician, and over 85% did not have children at the time of the survey. Regarding their career goals, over 50% anticipated a future in academic medicine, and over 65% anticipated being a specialist, but over 30% were still undecided. Despite being trainees at a critical stage in their careers, more than 50% had not received any formal career development planning advice, and more than 45% felt that they had not been given resources that addressed work-life balance even though over half were already struggling with it. The topics that survey respondents were most interested in were career development planning, work-life balance, and negotiation skills. Based on the responses, FWIM organized approximately 10-12 events annually, including workshops, lunch talks, and networking opportunities.

Although the focus of FWIM is equipping trainees early in their careers at a critical time, it has also fulfilled a need for community among women faculty. The Department of Medicine includes over 400 women faculty, fellows, and house staff, and previously lacked a formal outlet for women physicians across different divisions to come together and share their thoughts and knowledge. More specifically, FWIM serves as the central group responsible for organizing events, such as networking socials, that help bridge silos in a large department. FWIM has also leveraged social media, especially Twitter, to promote events, share articles and publications, and most importantly, increase the visibility of work being done by women at our institution; FWIM additionally sponsors women to attend career development conferences and supports students, residents, fellows, and faculty presenting at national conferences.

Overall, FWIM’s work has been received very positively as effectively meeting trainee needs. Feedback from postevent surveys designed by FWIM leadership has been collected since 2014 asking participants to evaluate their satisfaction with the event on a scale of 1 to 5, as well as short answer questions about what they appreciated or would change in the future. There was an average satisfaction rating of 4.7 out of 5 for workshops and 4.6 out of 5 for networking events. Narrative feedback has also been remarkably positive, including comments such as “[What I valued most from the workshop was] learning the language to advocate for myself” and “[One piece of advice I learned today I will implement is] to encourage others to speak up and assume positive intent.”

The positive outcomes measured suggest that FWIM has been a powerful intervention that effectively supports women trainees at a critical period in their careers. While the incoming class of 2014 was 23% women, the class of 2022 was 46% women. Furthermore, FWIM’s sustainability and growth since 2014 are also a testament to its feasibility and success. For example, FWIM recently created a trainee leadership development program that recruited a cohort of 12 residents and fellows in a longitudinal 8-month program. Additionally, new subcommittees have also been created: the Fellows Outreach Subcommittee to better engage fellows across various divisions in 2018 and the Department of Medicine Moms & Caregivers Subcommittee to provide support, advocacy, and education to women with caregiving responsibilities in 2020. Our findings are consistent with the existing literature, which has reported that programs expanding effective mentorship opportunities improve performance, increase self-assessed preparedness among residents prior to graduation, and impact trainee decision-making [14-16].

## Nearly a Decade of FWIM: Lessons Learned

Beyond our program, FWIM hopes to contribute to the greater community of women in medicine by serving as a framework for the development of similar trainee-focused programs both within and outside our institution. The establishment of FWIM has imparted several valuable insights about both the importance of FWIM and the factors that contributed to its subsequent success, enabling the program to support more than 110 trainees per year over the course of 8 years. This experience has been summarized into five points we recommend to other institutions seeking to pilot a similar program.

First, we recommend engaging early with support from leadership. At the time FWIM was created, the Department of Medicine chair championed FWIM, offering a platform for publicity and financial support. In fact, the chair was the driving force behind the creation of FWIM, and her unwavering advocacy encouraged participation from the entire department. Additionally, the budget secured administrative support and protected time for the director to lead and grow the initiative. Her clear support of FWIM’s mission set a culture change in motion; thus, we highly encourage taking the critical step to recruit stakeholder buy-in from leadership and colleagues when establishing a women’s group.

Second, we recommend developing a strategic plan and keeping a detailed record of the group’s activities for impact assessment. By hosting listening sessions and conducting a needs assessment, programming can be targeted with specific objectives to achieve. Records of these events ultimately serve as a measure of success and organizational health, which can be incredibly valuable when advocating for expansion, seeking stakeholder buy-in, and justifying the financial support mentioned earlier.

Third, we recommend recruiting representatives and advocates consisting of faculty, fellows, and house staff in different divisions, which facilitated outreach and networking between groups that had traditionally been disconnected. This evolved naturally from networking events as interested individuals self-selected as participants; more active recruitment efforts can also take place by seeking individuals with an interest in gender equity or graduate medical education. A similar model can be applied in large fractionated groups or between departments. These liaisons from different divisions are also a source of feedback in the form of needs assessments to refine programming.

Fourth, we recommend organizing collaborations with other groups as an opportunity to expand the group’s network. This also effectively lessens the financial burden of inviting guest speakers and, by cross-promoting events, reaches more people. For instance, FWIM often promotes conferences or events about diversity and inclusion from other organizations. FWIM also collaborates with medical students in the American Medical Women’s Association to host mentorship events that efficiently use a cascading mentorship model, the campus-wide faculty-based Academic Women’s Network, and other departments.

Finally, we recommend using resources such as the Group on Women in Medicine and Science (GWIMS) Toolkit for establishing a women in medicine and science group published by the Association of American Medical Colleges [17]. Other researchers have also synthesized strategies that can guide the establishment of a women’s affinity group and provide advice [18].

## Looking Forward

Gender inequities in medicine begin far before visible gaps in promotion, pay, and publishing develop. Thus, to address gender inequities, it is critical to proactively provide additional support to women trainees in addition to advocating for institutional and cultural changes. Trainee wellness has also become an important discussion over the past few years, and early intervention has been shown to affect trainees’ career goals and experience. As there is ever more need to enhance inclusiveness, representation, mentorship, allyship, and intersectionality in the field of medicine, we want to share the experience of FWIM to ignite discussion on innovative ways to foster a culture of care and equity. We hope to use this successful example of organizing programming designed for women trainees to inspire similar interventions at other institutions. Larger changes need to be implemented within the medical community to address gender bias, and the formation of groups such as FWIM can serve to support women as we advocate for ourselves and system-wide progress.

## Abbreviations

FWIM: Forum for Women in Medicine
GWIMS: Group on Women in Medicine and Science
